# Serum Gamma Glutamyltransferase Is Associated with 25-Hydroxyvitamin D Status in Elderly Patients with Stable Coronary Artery Disease

**DOI:** 10.3390/ijerph17238980

**Published:** 2020-12-02

**Authors:** Aleksander Danikiewicz, Bartosz Hudzik, Justyna Nowak, Joanna Kowalska, Iwona Zieleń-Zynek, Janusz Szkodzinski, Han Naung Tun, Barbara Zubelewicz-Szkodzinska

**Affiliations:** 1 Department of Nutrition Related Prevention, Faculty of Health Sciences in Bytom, Medical University of Silesia, 41-900 Bytom, Poland; adanikiewicz@sum.edu.pl (A.D.); jk.kwlsk@gmail.com (J.K.); izielen.zynek@gmail.com (I.Z.-Z.); bzubelewicz-szkodzinska@sum.edu.pl (B.Z.-S.); 2 Department of Cardiovascular Disease Prevention, Faculty of Health Sciences in Bytom, Medical University of Silesia, 41-900 Bytom, Poland; bartekh@mp.pl; 3 Third Department of Cardiology, Silesian Center for Heart Disease, Faculty of Medical Sciences in Zabrze, Medical University of Silesia, 41-800 Zabrze, Poland; janszkod@poczta.onet.pl; 4 Research and Clinical Working Groups, European Society of Cardiology, 06-903 Sophia Antipolis, France; annasxhan@gmail.com

**Keywords:** gamma glutamyltransferase, vitamin D, 25(OH)D, coronary artery disease

## Abstract

Background: No previous study has investigated the association between gamma glutamyltransferase (GGT) and vitamin D in patients with stable coronary artery disease (CAD). We investigated the cross-sectional associations between vitamin D status as assessed by serum 25(OH)D and GGT. Methods: 169 patients were enrolled. Study population was divided into three groups: 1: 25(OH)D < 10 ng/mL (*n* = 59); 2: 25(OH)D 10–20 ng/mL (*n* = 82), and 3: 25(OH)D > 20 ng/mL (*n* = 28). Based on a cut-off GGT value identified in ROC analysis, we also divided the study population to compare the following groups: GGT ≤19 (*n* = 66) and GGT >19 (*n* = 103). Results: GGT activity was the highest in vitamin D severely deficient patients and the lowest in vitamin D insufficient patients. GGT was inversely correlated with 25(OH)D concentrations (R = −0.23; *p* = 0.002). The receiver operating characteristics curve identified the discrimination threshold of GGT of >19 U/L in predicting vitamin D deficiency. Higher leukocyte and neutrophil counts and lower 25(OH)D concentration were found in patients with GGT > 19 U/L. Conclusions: We identified an interaction between declining 25(OH)D levels and rising GGT levels with increasing age, which resulted in an unfavorable 25(OH)D-to-GGT ratio in stable CAD patients. These results suggest that these changes might further contribute to a high cardiovascular risk in the elderly.

## 1. Introduction

Inflammation is a hallmark feature of atherogenesis. Experimental studies demonstrated the importance of innate immunity and the network of pro- and anti-inflammatory cytokines and growth factors in the formation and progression of atherosclerotic lesions within the arterial wall. This, in turn, results in systemic milieu that facilitate atherothrombotic events [1]. The generation of excess reactive oxygen species resulting from oxidative stress emerged as a critical element in the common pathway, leading to vascular injury and subsequently to atherosclerosis [2]. Reduced glutathione (GSH) is a small water-soluble tripeptide present in small quantities and one of the most important non-protein antioxidants in human cells. GSH protects by both directly scavenging oxidants and serving as the reductant for GSH-dependent antioxidant enzymes, such as glutathione peroxidase and glutathione reductase [3]. Attenuated GSH concentrations were implicated in the development of subclinical atherosclerosis [4]. Gamma glutamyltransferase (GGT) is a ubiquitous cell surface enzyme that cleaves extracellular glutathione (GSH). It increases the availability of amino acids, primarily cysteine, for intracellular GSH synthesis and has a pivotal role in maintaining GSH homeostasis and defense against oxidative stress. Measurement of serum GGT activity is widely used for the diagnosis of liver and obstructive biliary diseases and as an indicator of alcohol consumption [5]. Epidemiological studies suggest an association between GGT and coronary artery disease (CAD), a wide variety of cardiometabolic risk factors, e.g., hyperlipidemia, hypertension, diabetes, metabolic syndrome, systemic inflammation, and oxidative stress among others [5,6,7]. Studies demonstrated the presence of catalytically active GGT in atherosclerotic plaques and a correlation between GGT activity and indices of plaque instability, thereby suggesting direct involvement in the pathophysiology of atherosclerosis and related clinical events via promotion of pro-oxidant reactions by the enzyme [5,8,9].

Low and very low levels of vitamin D are very common in society worldwide [10]. Actually, serum 25-hydroxyvitamin D (25(OH)D) levels best indicate body vitamin D status, as they both manifest the dietary intake and cutaneous synthesis of vitamin D [11]. Its main function is to regulate bone metabolism and mineral homeostasis. Notwithstanding, many observational studies showed the association between vitamin D deficiency and risk of various cardiovascular disease (e.g., CAD, myocardial infarction, hypertension, heart failure, among others), but the biological mechanism of the causality remains to be elucidated [12,13,14,15].

Whilst many studies have been carried out on the association between liver conditions and vitamin D [16], no previous study has investigated the association between vitamin D status and GGT in patients with stable CAD. Given the paucity of evidence on the link between vitamin D status and GGT, we sought to obtain data which may help to address these research gaps. Therefore, we investigated the cross-sectional associations between vitamin D status as assessed by serum 25(OH)D and GGT.

## 2. Materials and Methods

The study was approved by the bioethics board at the Medical University of Silesia. It conforms to the Declaration of Helsinki. Seventy-eight patients with stable CAD were enrolled in an out-patient cardiology clinic. Detailed inclusion and exclusion criteria were previously published [17]. Stable CAD was defined as a history of documented myocardial infarction, prior coronary revascularization, chest pain with documented myocardial ischemia. Exclusion criteria included, among others, history of acute coronary syndrome within 12 months prior to enrollment or previous percutaneous and/or surgical revascularization within 12 months prior to enrollment, use of vitamin D/multivitamin/calcium supplements within 12 months prior to enrollment, cancer, chronic kidney disease—stage 3 or higher, uncontrolled thyroid dysfunction, liver dysfunction (including viral hepatitis, cholestatic jaundice with bilirubin concentration >1.5 mg/dL and/or alkaline phosphatase over the upper limit of normal; alanine transaminase (ALT) and aspartate aminotransferase (AST) over the upper limit of normal), calcium metabolic disorders, hormone replacement therapy, coexisting autoimmune disorders, acute infectious disease, chronic inflammatory disease, glucocorticoids and/or androgens therapy, and lack of patient consent to participate [17]. Arterial hypertension, hyperlipidemia, and diabetes mellitus were evaluated based on previous diagnosis according to medical records made according to the available guidelines [18,19,20].

Given seasonal variations in serum 25(OH)D concentrations, all blood samples were collected in the spring season. Venous blood samples were collected between 8–9 AM and separated by centrifugation at 4 °C, 1800× *g* for 15 min. Samples were then stored at −80 °C until further analysis. Serum 25-hydroxyvitamin D (25(OH)D) measurements were carried out using a commercial kit—Architect (Abbott Diagnostics, Lake Forest, IL, USA) 25OH Vitamin D chemiluminescence microparticle immunoassay (CMIA) in duplicate. Assay range was determined as 3.4–156.0 ng/mL. Measurements for each patient were made with the same kit to avoid inter-kit variability. The intra-assay coefficient of variation (% CV) was 2.7%, the inter-assay % CV was 3.5%. Precision error was <8% [17]. The test was standardized according to the Reference Measurement Procedure of the US National Institute of Standards and Technology. Serum 25(OH)D concentrations below 10 ng/mL were regarded as severe deficiency, those between 10–20 ng/mL were regarded as deficiency, and concentrations between 20–30 ng/mL were classified as insufficiency [21].

Serum GGT was measured through an enzymatic colorimetric assay using a COBAS INTEGRA 400 plus analyzer (Roche Diagnostics, GmbH, Mannheim, Germany). The minimum detectable threshold was 3 U/L and the measurement range was 3–1200 U/L. The intra-assay coefficient of variation (CV) was 1.5% and the inter-assay CV was 1.4%. The normal values considered for GGT were: 10–50 U/L in men and 10–38 U/L in women [22].

In total, 169 patients with stable CAD were enrolled. Based on serum 25(OH)D status, the study population wad divided into three groups [23]: group 1—25(OH)D concentration of less than 10 ng/mL (*n* = 59), group 2—25(OH)D concentration of 10–20 ng/mL (*n* = 82), and group 3—25(OH)D concentration of more than 20 ng/mL (*n* = 28). Based on a cut-off GGT value identified in the ROC analysis, we also divided the study population to compare the following groups: GGT ≤ 19 (*n* = 66) and GGT >19 (*n* = 103).

## 3. Statistical Analysis

Quantitative data are presented as medians and interquartile ranges (lower and upper quartiles) as most of the data (all but two) had a distribution other than normal. Qualitative data are presented as frequencies. The Shapiro–Wilk test was used to determine whether a random sample was normally distributed. Kruskal–Wallis ANOVA was used to test the differences between the three groups. The Chi-square test with Yates’ correction and Fisher’s exact test were used to compare categorical variables. Quantitative variables in the GGT ≤ 19 vs. GGT >19 analysis were compared using U Mann–Whitney test. The relationship between vitamin D and GGT was evaluated by Spearman’s rank correlation coefficient. A receiver operating characteristic (ROC) analysis was performed to assess the ability of GGT to predict 25(OH)D deficiency (defined as 25(OH)D concentration less than 20 ng/mL). Multivariate logistic regression models were chosen to evaluate the associations between GGT and 25[OH]D deficiency/insufficiency. Model 1 was adjusted for known risk factors linked to 25[OH]D deficiency/insufficiency (age, sex, weight, and waist circumference). Model 2 was further adjusted for variables with significant differences between the three groups (age, sex, weight, waist circumference, serum albumin, neutrophil count, ALT, and serum bilirubin).

## 4. Results

Baseline and clinical characteristics were similar irrespective of vitamin D status (Table 1).

GGT activity was the highest in the vitamin D severely deficient patients and the lowest in the vitamin D insufficient patients. Other laboratory results were similar (Table 2), except for albumin and neutrophil count, which were the highest in Group 3, and GGT, which was the lowest in Group 3.

GGT was inversely correlated with 25(OH)D concentrations (R = −0.23; *p* = 0.002). Moreover, GGT was inversely correlated with HDL cholesterol and lymphocyte count and positively correlated with white blood count and neutrophil count (Table 3).

The receiver operating characteristics curve identified the discrimination threshold of GGT of more than 19 U/L in predicting vitamin D deficiency (Table 4).

This cut-off had a high positive predictive value (PPV 88%). Subanalysis of the study population based on this cut-off showed higher leukocyte and neutrophil counts and lower 25(OH)D concentration in patients with GGT in the upper range of normal (Table 5).

**Figure 1:**
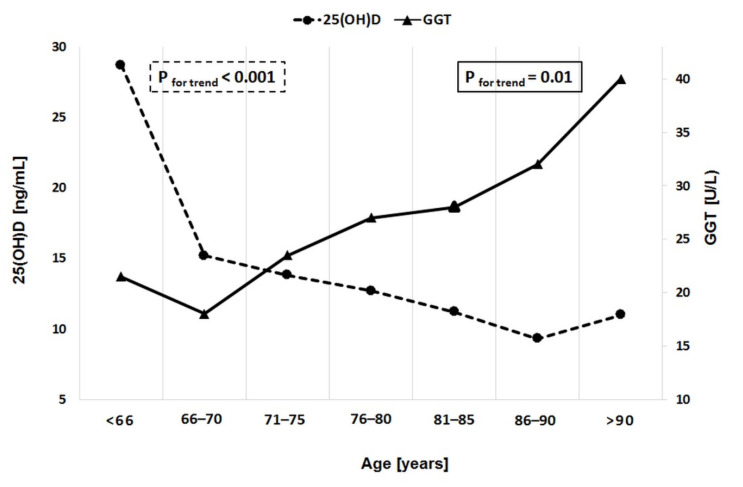
Age range-dependent changes in 25-hydroxyvitamin D (25(OHD)) and gamma glutamyltransfarase (GGT) levels.

In addition, the rate of more severe forms of vitamin D deficiency was more prevalent in patients with GGT greater than 19 U/L. With advancing age, a significant increase in GGT levels and decline in 25(OH)D levels were observed (Figure 1).

**Figure 2:**
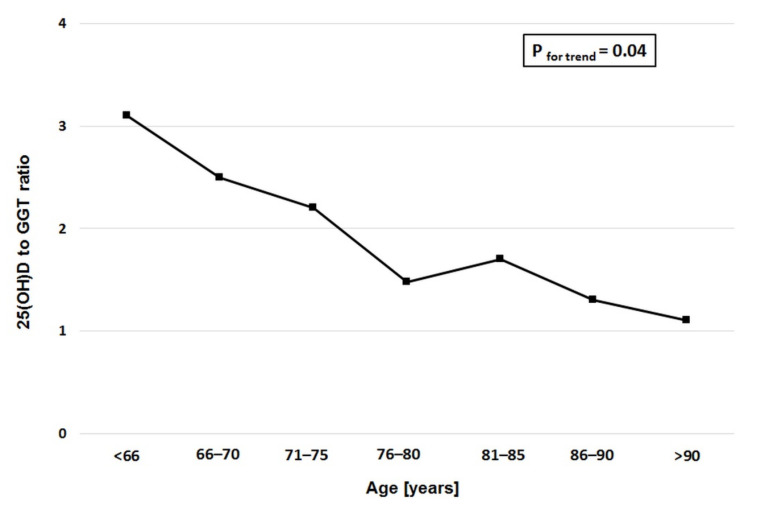
Age range-dependent changes in the 25-hydroxyvitamin D (25(OHD)) to gamma glutamyltransfarase (GGT) ratio.

Further analysis demonstrated a significant decline in the 25(OD)-to-GGT ratio (Figure 2).

GTT proved to be associated with the risk of 25[OH]D deficiency/insufficiency in unadjusted and adjusted models (Table 6).

## 5. Discussion

We have set out to determine the association between vitamin D and GGT in patients with stable CAD. There are five key findings of our study. First and foremost, GGT activity differs across the whole spectrum of vitamin D status (insufficiency, moderate deficiency, and severe deficiency), being the highest in the severely deficient patients and the lowest in insufficient patients. Second, there was a weak negative correlation between 25(OH)D and GGT. Moreover, GGT showed a negative correlation with HDL cholesterol and lymphocyte count and a positive correlation with white blood count and neutrophil count. In addition, GGT demonstrated a weak value in predicting vitamin D deficiency. Finally, more patients with GGT in the upper normal range had more severe forms of vitamin D deficiency.

Many studies implicate GGT as a potential biochemical marker for preclinical atherosclerosis. It was detected in atheromatous plaques of carotid and coronary arteries, triggering the oxidation of low-density lipoproteins (LDLs) [24]. GGT serves as a major source of intracellular support for the precursors of GSH, which is an important antioxidant [25]. Studies show that the elevated serum GGT activity can be a biomarker of increased oxidative stress in humans [26,27], which was shown to play a pivotal role in atherogenesis [24,26,27,28,29]. Demircan et al. reported that GGT levels were higher in patients with CAD than in the control group (38.7 ± 30.9 U/L versus 27.5 ± 17.5 U/L, *p* = 0.025) [30]. However, the serum GGT activity did not discriminate between the patients with 1-, 2-, and 3-vessel CAD. Notwithstanding, patients with acute coronary syndromes (ACS) had higher GGT activity compared to patients with stable CAD [30]. Regardless of the GGT pathogenetic considerations, GGT also displays prognostic value. Emdin et al. reported that serum GGT activity was related to cardiovascular mortality and nonfatal MI in patients with CAD and previous MI. They found that the prognostic value of GGT for cardiovascular mortality and nonfatal MI was present in patients with a history of MI [31]. Akpek et al. studied 425 patients with ST-elevation myocardial infarction (STEMI) and found that the in-hospital rate of major adverse cardiovascular events (MACE) increased with GGT tertiles. More importantly, they demonstrated in multivariate analysis that serum GGT activity was an independent predictor of in-hospital MACE (odds ratio (OR) 1.12, 95% confidence interval (CI) 1.01–1.33; *p* < 0.001) [32]. These results agree with those of others who confirmed the prognostic value of GGT in long-term follow-up [33,34].

In terms of vitamin D, its deficiency and insufficiency are common worldwide [10]. It has been attributed to greater sun protection, minimal outdoor sun exposure, and rise in the rate of overweight and obesity in the general population. In fact, all but one patient in the study population had vitamin D insufficiency or deficiency (25(OH)D concentration below 30 ng/mL). Moreover, 59 patients (34.9%) presented with severe vitamin D deficiency (25(OH)D concentration below 10 ng/mL), 82 patients (48.5%) presented with moderate vitamin D deficiency (25(OH)D concentration between 10–20 ng/mL), and 27 patients presented with vitamin D insufficiency (25(OH)D concentration between 20–30 ng/mL). In vitro experiments and animal studies demonstrated the expression of vitamin D receptors in endothelial cells, vascular smooth muscle, and cardiomyocytes. It was directly implicated in endothelium-mediated vasodilation, anticoagulant activity, and inhibition of the inflammatory response. Indirectly, it may favor the reduction of blood pressure, myocardial hypertrophy, and ventricular arrhythmias [35]. Vitamin D deficiency was linked to many cardiovascular conditions including, but not limited to, atherosclerosis, coronary artery disease, and myocardial infarction [36]. However, currently available evidence does not support cardiovascular benefits or disadvantages of vitamin D supplementation with the commonly used doses [37,38].

On the subject of vitamin D and GGT, we did not find any prior study addressing the link between GGT and vitamin D in stable CAD patients. The most interesting observation to emerge from our data comparison was the interaction between declining 25(OH)D levels and rising GGT levels with increasing age, which resulted in an unfavorable 25(OH)D-to-GGT ratio. We demonstrated an association between GGT and 25(OH)D deficiency/insufficiency both in unadjusted and adjusted models in elderly patients with stable CAD. Taken together, these results suggest that these changes might further contribute to a high cardiovascular risk in the elderly. The only evidence available on the link between vitamin D and GGT comes from non-alcoholic fatty liver disease (NAFLD). Barchetta et al. demonstrated that NAFLD patients have low 25(OH)D concentrations and elevated GGT activity (or in the upper range of normal limit) [39]. Moreover, they indicated that low 25(OH)D levels were associated with the presence of NAFLD independently of metabolic syndrome, diabetes, and insulin-resistance profile. He et al. examined the link between serum (25(OH)D) levels and serum liver enzymes in 24 229 US adults in a cross-sectional study. Their findings suggest a potential adverse effect of low 25(OH)D levels on human liver function (GGT OR 1.52 95%CI 1.32–1.75 *p* < 0.001) [40].

However, results from trials evaluating the effects of oral vitamin D supplementation on liver damage and liver enzymes (including GGT) in NAFLD are still inconsistent [41,42]. Nadreppor et al. examined the association between 25(OH)D and GGT, ALT, and alkaline phosphatase in 120 drug-naive individuals with no history of liver disease [43]. In addition, the effect of vitamin D supplementation (100,000 loading dose of cholecalciferol followed by 4000IU daily for 16 weeks) on hepatic enzymes was evaluated. They reported that 25(OH)D concentrations were not related to hepatic enzymes in drug-naive adults with no history of liver disease, and vitamin D supplementation had no effect on the serum levels of hepatic enzymes in vitamin D-deficient and overweight or obese, otherwise healthy individuals. Conversely, others noted a significant reduction in serum alkaline phosphatase and GGT with vitamin D and calcitriol supplementation from baseline levels [44]. Dabbaghmanesh et al. examined the role of vitamin D supplementation for the treatment of non-alcoholic fatty liver disease (NAFLD) [44]. In total, 106 NAFLD patients were randomized to receive calcitriol, vitamin D3, or placebo pearls for 12 weeks. Significant reductions in serum alkaline phosphatase and GGT were observed with vitamin D and calcitriol supplementation from baseline levels; however, no beneficial effect was noted when comparing vitamin D, calcitriol, and placebo groups at the end of the study. Available data support the hypothesis of the potential benefits of vitamin D supplementation in selected populations of NAFLD patients, such as those with shorter disease duration and mild to moderate liver damage [45].

In contrast to some studies, we found an inverse correlation between GGT and 25(OH)D (R = −0.23 *p* = 0.002). More interestingly, GGT had a significant moderate value in predicting vitamin D deficiency (AUC = 0.69) and severe vitamin D deficiency (AUC 0.63). A possible explanation for the association between GGT and vitamin D might be the inflammatory process. Accumulating evidence reveals that NAFLD as well as vitamin D deficiency are strongly related to inflammation. Cytokines and adipokines play a pivotal role in inflammatory processes [46,47,48,49]. In addition, these inflammatory mediators regulate various functions including metabolic energy balance, inflammation, and immune response. All of these actions may facilitate endothelial dysfunction, atherogenesis, and the progression of atherosclerosis [1]. Consistent with the literature, our research found significant, but relatively weak, positive correlations between GGT and leukocyte count and neutrophil count, and a relatively weak inverse correlation between GGT and lymphocyte count. High leukocytes, neutrophils, and GGT together with low lymphocytes and vitamin D could be a hallmark feature of the inflammatory and immune reactions in patients with stable CAD.

## 6. Conclusions

In summary, to the best of our knowledge, this is the first study assessing the association between GGT and vitamin D in stable CAD. Our study has identified a link between GGT and vitamin D in stable CAD patients. There is an inverse correlation between GGT and 25(OH)D. Moreover, serum GGT activity shows a weak to moderate positive correlation with leukocyte and neutrophil count and a weak, inverse correlation between GGT and lymphocyte count and HDL cholesterol. We identified an interaction between declining 25(OH)D levels and rising GGT levels with increasing age, which resulted in an unfavorable 25(OH)D-to-GGT ratio in stable CAD patients. Taken together, these changes might further contribute to a high cardiovascular risk in the elderly, although further large-scale studies are warranted to corroborate the causality relationship.

### Limitations

Our study needs to be viewed in light of its limitations. Given the cross-sectional study design, the analyses do not provide information about the possible causality of vitamin D deficiency and elevated GGT. The observational nature of the study cannot exclude a possible effect of any unmeasured factors on the observed associations. In addition, there is a seasonal change in 25(OH)D concentration; however, we avoided this problem by enrolling all patients in the spring season. Lower 25(OH)D concentrations during the winter season arise from less sun exposure and lower amounts of UV-B radiation reaching the skin, resulting in lower cutaneous vitamin D3 synthesis. More importantly, despite these limitations, we applied a strict set of exclusion criteria to obtain a homogenous population with respect to conditions and medical therapy that may have influenced GGT values and 25(OH)D concentrations; however, we did not screen for NAFLD. Finally, the relatively small number of patients enrolled might have rendered some differences insignificant. Further large-scale prospective cohort studies are warranted in the field of vitamin D and GGT. Intervention trials with vitamin D supplementation could perhaps provide more evidence on the causality of 25(OH)D and GGT levels.

## Figures and Tables

**Table 1:** Baseline clinical characteristics. Values are presented as medians (interquartile range) or *n* (percent).

	Group 1 25(OH)D < 10 ng/mL *n* = 59	Group 2 25(OH)D 10–20 ng/mL *n* = 82	Group 3 25(OH)D > 20 ng/mL *n* = 28	*p*
Age, years	77 (68–85)	76 (67–87)	75 (70–80)	0.1
Sex, men *n* (%)	13 (22.0)	35 (42.7)	10 (35.7)	0.0001
Arterial hypertension *n* (%)	42 (71.2)	69 (84.1)	24 (86.0)	0.6
Hyperlipidemia *n* (%)	37 (63.0)	48 (58.5)	16 (57.1)	0.8
Diabetes mellitus *n* (%)	16 (27.1)	23 (28.0)	11 (63.3)	0.4
Prior myocardial infarction *n* (%)	3 (5.1)	15 (18.3)	8 (39.2)	0.5
BMI	25 (23–28)	29 (24–31)	27 (24–30)	0.1

BMI—body mass index.

**Table 2:** Laboratory findings. Values are presented as medians (interquartile range).

	Group 1 25(OH)D < 10 ng/mL *n* = 59	Group 2 25(OH)D 10–20 ng/mL *n* = 82	Group 3 25(OH)D > 20 ng/mL *n* = 28	*p*
Leucocytes (10^3^/mm^3^)	6.4 (4.8–8.6)	6.8 (5.5–8.4)	6.1 (4.7–6.7)	0.1
Erythrocytes (10^6^/mm^3^)	4.1 (3.8–4.6)	4.3 (3.9–4.5)	4.1 (3.9–4.3)	0.5
Lymphocytes (10^3^/mm^3^)	2.0 (1.5–3.0)	2.3 (2.0–3.1)	2.0 (1.9–2.4)	0.7
Neutrophils (10^3^/mm^3^)	4.0 (2.6–5.3)	3.8 (3.0–4.8)	2.7 (2.2–3.9)	0.04
Hemoglobin (g/dL)	12.6 (12.1–13.8)	12.7 (11.7–13.4)	12.4 (11.3–13.1)	0.2
Hematocrit (%)	38 (36–42)	39 (36–41)	38 (37–39)	0.3
Platelets (10^3^/mm^3^)	220 (173–262)	205 (168–248)	225 (204–277)	0.1
Total cholesterol (mmol/L)	4.3 (3.8–5.5)	4.4 (3.5–5.3)	4.3 (3.7–5.1)	0.7
HDL cholesterol (mmol/L)	1.5 (1.0–1.9)	1.5 (1.2–1.7)	1.2 (1.1–1.3)	0.1
LDL cholesterol (mmol/L)	2.3 (1.3–3.6)	2.6 (1.7–3.3)	2.5 (1.8–3.2)	0.8
Triglycerides (mmol/L)	1.0 (0.9–1.4)	1.1 (0.9–1.4)	1.1 (0.9–1.9)	0.8
Serum creatinine (μmol/L)	70 (60–90)	76 (66–100)	75 (66–88)	0.1
Aspartate aminotransferase (AST) (U/L)	18 (15–20)	18 (16–26)	16 (14–19)	0.1
Alanine aminotransferase (ALT) (U/L)	16 (10–22)	15 (11–20)	13 (11–17)	0.4
Bilirubin (mmol/L)	12.3 (9.5–18.1)	9.7 (8.4–12.0)	10.1 (8.2–13.5)	0.08
GGT (U/L)	27 (23–42)	20 (17–40)	16 (11–25)	0.04
Total protein (g/L)	60.9 (57.2–62.5)	64.5 (56.0–67.3)	62.1 (58.0–64.7)	0.07
Albumin (mg/mL)	36 (32–38)	37 (31–39)	42 (38–46)	0.04
25(OH)D (ng/mL)	8.4 (7.1–9.3)	13.4 (12.1–15.8)	23.4 (21.3–25.2)	<0.001

25(OH)D—25-hyroxyvitamin D; GGT—gamma glutamyltransferase; HDL—high-density lipoprotein; LDL—low-density lipoprotein.

**Table 3:** Correlations between gamma glutamyltranferase and clinical features and laboratory results.

	Gamma Glutamyltransferase	
	Spearman R	*p*
Weight	0.20	0.008
Waist-to-hip ratio	0.20	0.008
Waist-to-height ratio	0.15	0.04
25(OH)D	−0.23	0.002
HDL cholesterol	−0.24	0.002
Alanine aminotransferase (ALT)	0.26	0.0005
Bilirubin	0.25	0.0002
Hemoglobin	0.20	0.01
Leukocytes	0.23	0.003
Neutrophils	0.22	0.004
Lymphocytes	−0.15	0.05

**Table 4:** Receiver operating characteristics curves identifying the discrimination thresholds of gamma glutamyltransferase for vitamin D deficiency and severe vitamin D deficiency.

	Cut-Off	AUC	95% CI	Sensitivity	Specificity	PPV	NPV	*p*
Vitamin D Deficiency								
GGT	>19	0.69	0.53–0.77	63%	64%	88%	28%	0.04
Severe Vitamin D Deficiency								
GGT	>21	0.63	0.51–0.74	81%	56%	41%	86%	0.04

AUC—area under the curve; PPV—positive predictive value; NPV—negative predictive value; GGT—gamma glutamyltransferase.

**Table 5:** Clinical and laboratory features based on GGT status. Values are presented as medians (interquartile range) or *n* (percent).

	GGT ≤ 19 (*n* = 66)	GGT > 19 (*n* = 103)	*p*
Age, years	77 (65–85)	77 (68–82)	0.6
Sex, men *n* (%)	23 (34.8)	35 (34.0)	0.9
Systemic hypertension *n* (%)	51 (77.2)	84 (81.5)	0.7
Hyperlipidemia *n* (%)	40 (60.6)	61 (59.2)	0.5
Diabetes mellitus *n* (%)	17 (25.7)	33 (32.0)	0.2
Prior myocardial infarction *n* (%)	10 (15.2)	16 (15.5)	0.9
BMI	26 (23–30)	27 (24–31)	0.4
Leucocytes (10^3^/mm^3^)	5.7 (5.1–7.0)	7.0 (5.6–8.9)	0.01
Erythrocytes (10^6^/mm^3^)	4.2 (3.9–4.4)	4.2 (3.9–4.7)	0.6
Lymphocytes (10^3^/mm^3^)	2.1 (1.9–2.6)	2.2 (1.8–3.0)	0.4
Neutrophils (10^3^/mm^3^)	3.1 (2.5–4.6)	4.0 (2.9–5.5)	0.01
Hemoglobin (g/dL)	12.4 (11.6–13.1)	12.7 (12.0–13.8)	0.2
Hematocrit (%)	38 (36–40)	39 (37–42)	0.2
Platelets (10^3^/mm^3^)	216 (190–253)	209 (160–279)	0.2
Total cholesterol (mmol/L)	4.4 (3.5–5.4)	4.4 (3.7–5.4)	0.7
HDL cholesterol (mmol/L)	1.5 (1.2–1.7)	1.3 (1.0–1.8)	0.1
LDL cholesterol (mmol/L)	2.5 (1.7–3.4)	2.5 (1.6–3.2)	0.7
Triglycerides (mmol/L)	1.0 (0.9–1.4)	1.1 (1.0–1.6)	0.7
Serum creatinine (μmol/L)	73 (65–91)	75 (62–92)	0.7
Aspartate aminotransferase (AST) (U/L)	18 (16–20)	18 (15–23)	0.8
Alanine aminotransferase (ALT) (U/L)	14 (11–16)	17 (11–22)	0.02
Bilirubin (mmol/L)	9.9 (8.2–11.1)	10.90 (8.4–19.2)	0.02
Total protein (g/L)	60.5 (57.3–64.0)	60.9 (58.0–68.4)	0.7
Albumin (mg/mL)	34 (33–38)	34 (31–38)	0.2
25(OH)D (ng/mL)	13.8 (11.0 –21.0)	10.9 (8.4–15.2)	0.0001
• Vitamin D insufficiency (25(OH)D 20–30 ng/mL) *n* (%)	10 (15.2)	18 (17.1)	
• Moderate vitamin D deficiency (25(OH)D 10–20 ng/mL)	38 (57.7)	44 (42.7)	0.0002
• Severe vitamin D deficiency (25(OH)D < 10 ng/mL) *n* (%)	18 (27.1)	41 (39.8)	

**Table 6:** GGT and the risk of 25[OH]D deficiency/insufficiency.

25[OH]D Serum Level < 30 ng/mL, Odds Ratio (95% Confidence Intervals), *p*-Value		
Unadjusted Model 1 Model 2	1.07 (1.02–1.11) 1.06 (1.02–1.12) 1.05 (1.01–1.11)	0.004 0.01 0.04
Model 1, adjusted for age, sex, weight, waist circumference Model 2 adjusted for age, sex, weight, waist circumference, serum albumin, neutrophil count, ALT, serum bilirubin		

ALT—Alanine aminotransferase, GGT—gamma glutamyltransferase
